# Hierarchical Heterostructure of ZnO@TiO_2_ Hollow Spheres for Highly Efficient Photocatalytic Hydrogen Evolution

**DOI:** 10.1186/s11671-017-2304-5

**Published:** 2017-09-13

**Authors:** Yue Li, Longlu Wang, Jian Liang, Fengxian Gao, Kai Yin, Pei Dai

**Affiliations:** 1 School of Materials and Chemical Engineering, Henan University of Engineering, Zhengzhou, 451191 Henan People’s Republic of China; 2 State Key Laboratory of Chemo/Biosensing and Chemometrics, Hunan University, Changsha, 410082 People’s Republic of China

**Keywords:** ZnO, TiO_2_, Hollow sphere, Hierarchical, Heterojunction, Hydrogen production

## Abstract

The rational design and preparation of hierarchical nanoarchitectures are critical for enhanced photocatalytic hydrogen evolution reaction (HER). Herein, well-integrated hollow ZnO@TiO_2_ heterojunctions were obtained by a simple hydrothermal method. This unique hierarchical heterostructure not only caused multiple reflections which enhances the light absorption but also improved the lifetime and transfer of photogenerated charge carriers due to the potential difference generated on the ZnO–TiO_2_ interface. As a result, compared to bare ZnO and TiO_2_, the ZnO@TiO_2_ composite photocatalyst exhibited higher hydrogen production rated up to 0.152 mmol h^−1^ g^−1^ under simulated solar light. In addition, highly repeated photostability was also observed on the ZnO@TiO_2_ composite photocatalyst even after a continuous test for 30 h. It is expected that this low-cost, nontoxic, and readily available ZnO@TiO_2_ catalyst could exhibit promising potential in photocatalytic H_2_ to meet the future fuel needs.

## Background

Hydrogen (H_2_), one of the most important clean and sustainable energy, has been regarded as a promising alternative energy for meeting future fuel needs [1–5]. Since the discovery of photoelectrochemical (PEC) water-splitting system by Fujishima and Honda in the 1970s [6], the production of H_2_ based on TiO_2_ semiconductor photocatalysts using sunlight has attracted increasing attention. However, the practical application of single bare TiO_2_ in the industry is still a challenge due to the high-rate recombination of photogenerated electrons and holes at the surface of TiO_2_ results in a low quantum efficiency. To date, many efforts have been made to design TiO_2_-based composite photocatalysts to solve the above issues, such as coupling with another semiconductor, doping transition metal ions or nonmetal atoms, and so on [7–9]. In particular, the formation of semiconductor–semiconductor heterojunctions with matching band potentials is an effective way to prevent the charge recombination and increase the lifetime of the charge carriers [10–12].

Among the various semiconductors, ZnO is also extensively studied because of its identical properties of TiO_2_ with non-toxicity, cheapness, high efficiency, and chemical stability [13, 14]. Since the conduction band (CB) and valence band (VB) of ZnO lie above those of TiO_2_, the photogenerated electrons in ZnO will be transferred to TiO_2_ once a heterojunction was formed between TiO_2_ and ZnO. This kind of ZnO@TiO_2_ composite heterojunction will benefit for the separation of photogenerated electron–hole pairs, thus leading more electrons accumulated on the TiO_2_ which will react with H_2_O to generate H_2_ [15–17].

In addition to the above we have discussed, geometric shapes and morphologies of the photocatalysts also heavily influence the hydrogen evolution reaction (HER) performance [18–20]. It is has been reported that the diffractions on the hollow spheres and the multiple reflections due to the shell structure would enhance the effectiveness of light utilization [21]. For example, Li’s group prepared hydrogenated cage-like titania hollow spheres exhibited much higher HER activities than solid structure [22]. Beyond that, the spherical hollow structures have the advantages of large specific surface area, reduced transport lengths for charge carriers, and good chemical and thermal stability, which all contribute to the excellent photocatalytic ability [23]. However, most of the research has focused on the preparation of composite hollow spheres by doping transition element, such as Ce–ZnO [24], Ni–ZnO [25], Ag–TiO_2_ [26], Au–TiO_2_ [27], and so on. To the best of our knowledge, few studies reported on the synthesis of closed, complete, and intact hollow spheres composed of mixed metal oxides porous particles. Even so, most of these composites are applied in photocatalytic degradation of organic pollutants but not in the photocatalytic hydrogen production.

In this paper, we reported a facile method to synthesize hierarchically porous ZnO@TiO_2_ composite hollow microspheres and applied them in the photocatalytic H_2_. The hollow spheres enhanced the light absorption by multiple reflections, at the same time, the lifetime and transfer rate of photogenerated charge carriers were also improved due to the potential difference generated on the ZnO–TiO_2_ interface. The result showed that the ZnO@TiO_2_ composite photocatalyst exhibited enhanced H_2_ evolution rate, compared to the bare ZnO and TiO_2_. In addition, the mechanism of the photocatalytic H_2_ on the ZnO@TiO_2_ composite hollow spheres was discussed in detail.

## Methods

### Synthesis of the Hierarchical ZnO@TiO_2_ Hollow Spheres

The preparation of ZnO@TiO_2_ composites was based on a very facile one-step template-free hydrothermal method at ambient conditions. In a typical procedure, 0.015 mol of Ti(SO_4_)_2_, 0.015 mol of Zn(NO_3_)_2_·6H_2_O, 0.015 mol of NH_4_F, and 0.06 mol of CO(NH_2_)_2_ were added to a beaker with 50 mL deionized water. After stirring for 60 min, the mixture solution was transferred into a Teflon-lined stainless steel autoclave and heated in an electric oven at 180 °C for 12 h. After that, the white precipitate was thoroughly washed with ethanol four times and then dried at 60 °C for 12 h to obtain ZnO@TiO_2_ heterostructures. For comparison, bare TiO_2_ and ZnO were prepared under the same conditions.

### Synthesis of Pt–ZnO@TiO_2_ Samples

In a typical synthesis process of Pt–ZnO@TiO_2_ samples, the ZnO@TiO_2_ hollow spheres were put into a container containing 10 vol% triethanolamine and H_2_PtCl_6_ solution. Then, the system was bubbled with nitrogen for 30 min to remove the air. Finally, the Pt was in situ photodeposited on the ZnO@TiO_2_ hollow spheres under a full arc light irradiation (*λ* > 300 nm) for 2 h. The Pt content can be tuned by the concentration of H_2_PtCl_6_ and the reaction time, which was determined by inductively coupled plasma (ICP, PE5300DV).

### Characterization

The morphology of ZnO@TiO_2_ heterostructures was characterized via field emission scanning electron microscope (FESEM, Hitachi, Japan), transmission electron microscopy (TEM, Tecnai F20), high-angle annular dark field scanning TEM (STEM, Tecnai F20), and high-resolution TEM (HRTEM, Tecnai F20). The energy-dispersive X-ray spectroscopy (EDS) mapping images were captured on a Tecnai G2 F20 S-TWIN atomic resolution analytic microscope. The crystal phase properties of the samples were characterized using an X-ray diffractometer with Cu–K radiation (XRD, M21X, MAC Science Ltd., Japan). The BET specific surface areas were measured on Belsorp-mini II analyzer (Japan).

### Photoelectrochemical Measurements

Photocurrent studies were performed on a CHI 660D electrochemical workstation, using a three-electrode configuration where fluorine-doped tin oxide (FTO) electrodes deposited with the samples as working electrode, Pt as counter electrode, and a saturated calomel electrode (SCE) as reference. The electrolyte was 0.35 M/0.25 M Na_2_S–Na_2_SO_3_ aqueous solution. For the fabrication of the working electrode, 0.25 g of the sample was grinded with 0.06 g polyethylene glycol (PEG, molecular weight 20,000) and 0.5 mL ethanol to make a slurry. Then, the slurry was spread onto a 1 × 4 cm FTO glass by the doctor blade technique and then allowed to dry in air. A 300 W xenon arc lamp served as a simulated solar light irradiation source (Perfectlight, PLS-SXE 300C, Beijing, China). The incident light intensity was tuned to be 100 mW/cm^2^ measured by NOVA Oriel 70260 with a thermodetector.

### Photocatalytic Hydrogen Production Tests

Photocatalytic hydrogen production experiments were performed in a sealed quartz flask at ambient temperature and under atmospheric pressure. A 300 W xenon arc lamp (Perfect light, PLS-SXE 300C, Beijing, China) was used as the light source to trigger the photocatalytic reaction. The evolved H_2_ were collected and online-analyzed by a H_2_-solar system (Beijing Trusttech Technology Co., Ltd.) with a gas chromatogram equipped with a thermal conductivity detector (TCD), 5A molecular sieve column, and nitrogen as the carrier gas. All photocatalytic experiments over 100 mg photocatalyst were performed in an aqueous solution containing H_2_O (80 mL) and alcohol (20 mL). Prior to irradiation, the system was deaerated by bubbling nitrogen for 15 min. During the photocatalytic reaction, the reactor was tightly sealed to avoid gas exchange.

## Results and Discussion

**Fig. 1:**
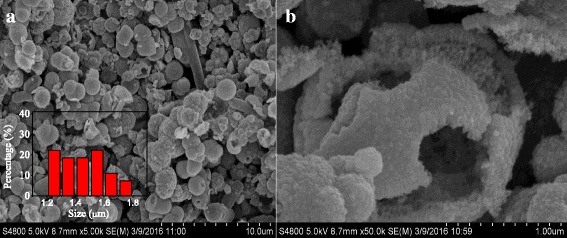
**a** A low-magnified SEM image of ZnO@TiO_2_ hollow spheres; the inset shows the statistical analysis of diameter distribution of the samples. **b** A high-magnified SEM image of a single broken ZnO@TiO_2_ sphere

**Fig. 2:**
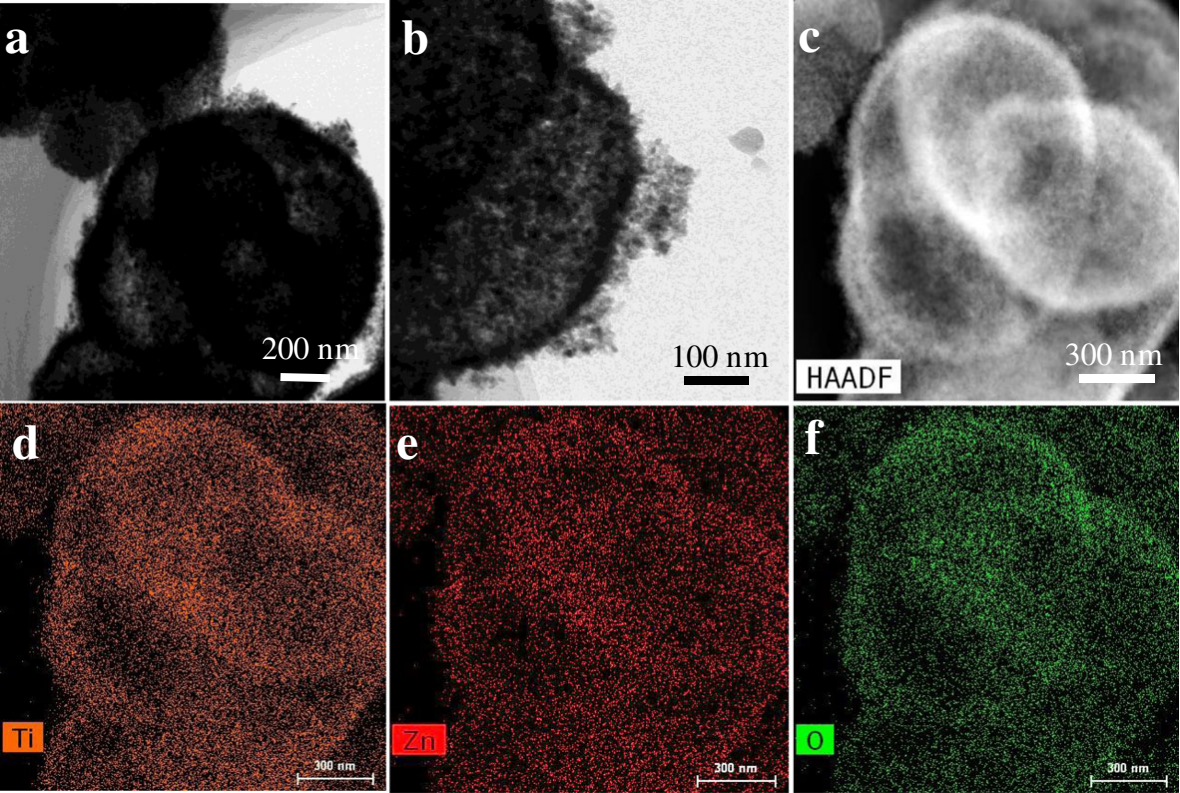
**a** TEM, **b** magnified TEM, and **c** STEM images of ZnO@TiO_2_ hollow spheres; Corresponding EDS elemental mappings of **c** indicating the uniform distribution of **d** Ti, **e** Zn, and **f** O, respectively

The size and morphology of the as-prepared ZnO@TiO_2_ hollow spheres were displayed in Fig. 1. Figure 1a shows the sample has a uniform spherical morphology with a mean diameter about 1.45 μm according to the nanoparticle size distribution (inset of Fig. 1a). Figure 1b reveals a single broken sphere, indicating that the prepared sample is a hollow structure composed of small particles. TEM image was further used to confirm the structure of the ZnO@TiO_2_ hollow spheres. The color change of the ZnO@TiO_2_ spheres at the center and the outside realm was dark and bright, respectively, confirming the ZnO@TiO_2_ spheres were hollow structure (Fig. 2a). A high-magnified view in Fig. 2b also depicts the surface of the hollow spheres was rough which were constructed by nanoparticles subunits, as a result in the formation of the hierarchical heterostructure of ZnO@TiO_2_ hollow spheres. The elemental maps in Fig. 2(d–f) were used to confirm the elemental distribution in the ZnO@TiO_2_ hollow spheres. It can be seen that the Zn, Ti, and O were uniformly distributed in ZnO@TiO_2_ hollow spheres.

**Fig. 3:**
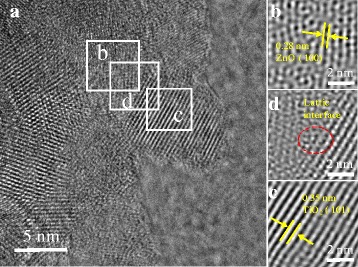
**a** HRTEM images of ZnO@TiO_2_ hollow spheres. **b**, **c**, and **d** are amplified HRTEM images of designated square part in **a**, indicating ZnO, TiO_2_, and ZnO@TiO_2_ heterojunctions, respectively

HRTEM images in Fig. 3 verified the heterojunction structure of ZnO@TiO_2_ hollow spheres. The selected areas in Fig. 3a marked by white square were magnified in Fig. 3b–d, corresponding to ZnO, TiO_2_, and ZnO@TiO_2_ heterojunction. The lattice spacing distances of 0.28 and 0.35 nm were corresponding to the (100) planes of wurtzite ZnO and (101) planes of the anatase TiO_2_, respectively, as shown in Fig. 3b, c. Figure 3d shows a clear transition from wurtzite ZnO phase to anatase TiO_2_ phase, which confirmed the heterojunction was formed at the interface between ZnO and TiO_2_. Such heterojunction structure can greatly promote the photoexcited electron transfer for enhanced photocatalytic activity.

**Fig. 4:**
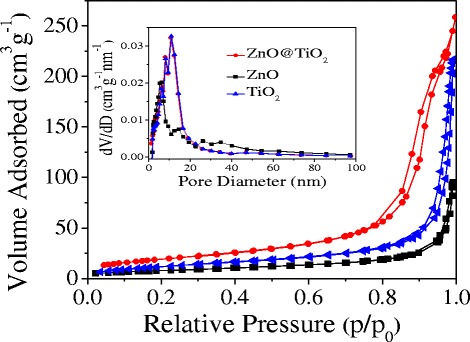
N_2_ adsorption–desorption isotherms and the inset show the corresponding pore size distribution curves

The pore structure properties of ZnO, TiO_2_, and ZnO@TiO_2_ samples were further determined by the N_2_ adsorption–desorption isotherms and corresponding Barrett–Joyner–Halenda (BJH) pore size distribution plots (Fig. 4). All the samples showed a type IV isotherm with a hysteresis loop at a high relative pressure (*P*/*P*_0_ > 0.7), demonstrating the existence of mesoporous structures according to International Union of Pure and Applied Chemistry (IUPAC) classification. The inset of Fig. 4 is BJH pore size distribution plots, which further indicated that all the samples have the mesoporous structures. Meanwhile, the calculated BET surface areas of the ZnO@TiO_2_ microsphere was about 102 m^2^ g^−1^, which was much larger than that of ZnO (23 m^2^ g^−1^) and TiO_2_ (35 m^2^ g^−1^). It can be concluded the introduction of ZnO into TiO_2_ to form the ZnO@TiO_2_ hollow spheres could increase the surface areas greatly, although all the samples have the mesoporous structures. The higher surface areas of ZnO@TiO_2_ hollow spheres would provide more sites for enhanced catalytic H_2_ performance.

**Fig. 5:**
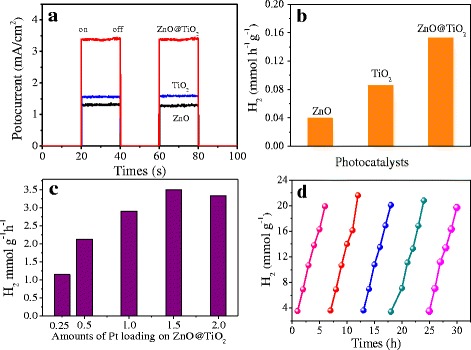
**a** Photocurrent responses and **b** photocatalytic H_2_ evolution of bare ZnO, bare TiO_2_, and ZnO@TiO_2_ heterojunctions. **c** Photocatalytic H_2_ evolution over Pt–ZnO@TiO_2_ heterojunctions composites with different weight ratios of Pt. **d** Photocatalytic stability of ZnO@TiO_2_ hollow spheres. All the measurement was carried out under a simulated solar light irradiation source with intensity of 100 mW/cm^2^

The photocatalytic ability of the as-prepared samples was evaluated by photocurrent and photocatalytic H_2_ tests. As shown in Fig. 5a, the ZnO@TiO_2_ hollow spheres yielded the highest photocurrent density of 3.38 mA/cm^2^, which was more than 2.61, 2.17 times higher than that of ZnO and TiO_2_, respectively. These results mean the stronger ability of producing charge carriers and improved separation efficiency of ZnO@TiO_2_ hollow spheres. As excepted, the hydrogen production rate of ZnO@TiO_2_ hollow spheres reached to 0.152 mmol h^−1^ g^−1^, higher than the 0.039 mmol h^−1^ g^−1^ of ZnO and 0.085 mmol h^−1^ g^−1^ of TiO_2_ (Fig. 5b). Pt, as a very high-efficiency noble metal cocatalyst, has been widely used for H_2_ evolution reaction in the reported literature [8, 11]. A series of Pt–ZnO@TiO_2_ with different Pt contents were prepared and compared in Fig. 5c. It was shown that the loading of Pt onto ZnO@TiO_2_ hollow spheres could significantly enhance the H_2_ evolution activity and the sample with 1.5 at % Pt exhibiting the highest H_2_ evolution rate. Figure 5d shows that the ZnO@TiO_2_ hollow spheres still retained its original photocatalytic activity without noticeable degradation in the five reaction cycles for 30 h, which demonstrates the exceptional photocatalytic stability.

**Fig. 6:**
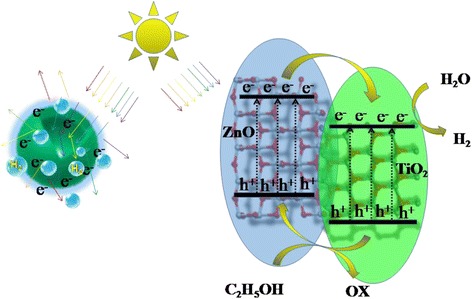
Schematic illustration of the proposed HER mechanism of ZnO@TiO_2_ hollow spheres

A photocatalytic mechanism was proposed for the improved HER activity of the ZnO@TiO_2_ hollow spheres, as shown in Fig. 6. Under simulated solar light irradiation, the electrons of both ZnO and TiO_2_ were excited from their valence bands (VB) to their conduction bands (CB). Since the conduction band (CB) and valence band (VB) of ZnO were more positive than those of TiO_2_, the photogenerated electrons transferred from ZnO to TiO_2_ through the intimate interfacial contacts [16]. Then, the more accumulated electrons on the TiO_2_ reacted with H_2_O for generating H_2_ for the higher photocatalytic H_2_ rate (as shown on the right of Fig. 6). At the same time, the photogenerated holes in the VB of TiO_2_ migrated to ZnO, which were trapped by the sacrificial agent to keep the thermodynamical balance. Additionally, the hierarchical hollow spheres benefit for light scatter and multiple reflections among ZnO@TiO_2_ composite photocatalyst, which would enhance the effectiveness of light utilization [10, 21, 22]. Thus, more free electrons and holes would be generated due to the increased effective photon path length [21, 22], leading to a higher HER efficiency (as shown in the left of Fig. 6).

## Conclusions

In summary, the hierarchical heterostructure of ZnO@TiO_2_ hollow spheres has been successfully prepared via a simple hydrothermal method. Compared to bare ZnO and TiO_2_, the ZnO@TiO_2_ composite photocatalyst exhibited high hydrogen production rated up to 0.152 mmol h^−1^ g^−1^ under simulated solar light. It is believed that hierarchical heterostructure increased the surface area which proving more active sites for effective HER and simultaneously improved the lifetime and transfer of photogenerated charge carriers due to the potential difference generated on the ZnO–TiO_2_ interface. Moreover, the ZnO@TiO_2_ composite photocatalyst exhibited good durability even after being reused five times. This work demonstrated a good prospect for photocatalytic H_2_ evolution from water based on the rational use and preparation of high activity, inexpensive, and chemical stability of ZnO and TiO_2_.

## References

[1] Zhou W, Li W, Wang JQ (2014). Ordered mesoporous black TiO_2_ as highly efficient hydrogen evolution photocatalyst. J Am Chem Soc.

[2] Li Y, Wang LL, Zhang SQ (2017). Cracked monolayer 1T MoS_2_ with abundant active sites for enhanced electrocatalytic hydrogen evolution. Catal Sci Technol.

[3] Zhang Y, Fu LJ, Shu Z (2014). Substitutional doping for aluminosilicate mineral and superior water splitting performance. Nanoscale Res Lett.

[4] Zhao L, Jia J, Yang Z (2017). One-step synthesis of CdS nanoparticles/MoS_2_ nanosheets heterostructure on porous molybdenum sheet for enhanced photocatalytic H_2_ evolution. Appl Catal B Environ.

[5] Li Y, Wang LL, Cai T (2017). Glucose-assisted synthesize 1D/2D nearly vertical CdS/MoS_2_ heterostructures for efficient photocatalytic hydrogen evolution. Chem Eng J.

[6] Fujishima A, Honda K (1972). Electrochemical photolysis of water at a semiconductor electrode. Nature.

[7] Park JH, Kim S, Bard AJ (2006). Novel carbon-doped TiO_2_ nanotube arrays with high aspect ratios for efficient solar water splitting. Nano Lett.

[8] Loget G, Schmuki P (2014). H_2_ mapping on Pt-loaded TiO_2_ nanotube gradient arrays. Langmuir.

[9] Zhou W, Yin Z, Du Y (2013). Synthesis of few-layer MoS_2_ nanosheet-coated TiO_2_ nanobelt heterostructures for enhanced photocatalytic activities. Small.

[10] Wang LL, Duan X, Wang G (2016). Omnidirectional enhancement of photocatalytic hydrogen evolution over hierarchical “cauline leaf” nanoarchitectures. Appl Catal B Environ.

[11] Park H, Kim YK, Choi W (2011). Reversing CdS preparation order and its effects on photocatalytic hydrogen production of CdS/Pt-TiO_2_ hybrids under visible light. J Phys Chem C.

[12] Liu CB, Wang LL, Tang YH et al (2015) Vertical single or few-layer MoS_2_ nanosheets rooting into TiO_2_ nanofibers for highly efficient photocatalytic hydrogen evolution. Appl Catal B Environ 164:1–9

[13] Zhang B, Wang F, Zhu C (2015). A facile self-assembly synthesis of hexagonal ZnO nanosheet films and their photoelectrochemical properties. Nano-Micro Lett.

[14] Kayaci F, Vempati S, Ozgit-Akgun C (2015). Transformation of polymer-ZnO core–shell nanofibers into ZnO hollow nanofibers: intrinsic defect reorganization in ZnO and its influence on the photocatalysis. Appl Catal B Environ.

[15] Li X, Lv K, Deng K (2009). Synthesis and characterization of ZnO and TiO_2_ hollow spheres with enhanced photoreactivity. Mater Sci Eng B.

[16] Agrawal M, Gupta S, Pich A (2009). A facile approach to fabrication of ZnO–TiO_2_ hollow spheres. Chem Mater.

[17] Wang Y, Zhu S, Chen XY (2014). One-step template-free fabrication of mesoporous ZnO/TiO_2_ hollow microspheres with enhanced photocatalytic activity. Appl Surf Sci.

[18] He H, Lin J, Fu W (2016). MoS_2_/TiO_2_ edge-on heterostructure for efficient photocatalytic hydrogen evolution. Adv Energy Mater.

[19] Wang L, Li Y, Liu Y (2017). Reduced graphene oxide@TiO_2_ nanorod@reduced graphene oxide hybrid nanostructures for photoelectrochemical hydrogen production. Micro Nano Lett.

[20] Wang L, Liu X, Luo J (2017). Self-optimization of the active site of molybdenum disulfide by an irreversible phase transition during photocatalytic hydrogen evolution. Angew Chem Int Ed.

[21] Kondo Y, Yoshikawa H, Awaga K (2008). Preparation, photocatalytic activities, and dye-sensitized solar-cell performance of submicron-scale TiO_2_ hollow spheres. Langmuir.

[22] Wang Y, Cai J, Wu M (2016). Hydrogenated cagelike titania hollow spherical photocatalysts for hydrogen evolution under simulated solar light irradiation. ACS Appl Mater Interfaces.

[23] Zhang C, Zhou Y, Zhang Y (2017). Double-shelled TiO_2_ hollow spheres assembled with TiO_2_ nanosheets. Chem Eur J.

[24] Jiang J, Zhang K, Chen X (2017). Porous Ce-doped ZnO hollow sphere with enhanced photodegradation activity for artificial waste water. J Alloy Compd.

[25] Wang Y, Liu T, Huang Q (2016). Synthesis and their photocatalytic properties of Ni-doped ZnO hollow microspheres. J Mater Res.

[26] Xiang Q, Yu J, Cheng B (2010). Microwave-hydrothermal preparation and visible-light photoactivity of plasmonic photocatalyst Ag-TiO_2_ nanocomposite hollow spheres. Chem-Asian J.

[27] Dinh CT, Yen H, Kleitz F (2014). Three-dimensional ordered assembly of thin-shell Au/TiO_2_ hollow nanospheres for enhanced visible-light-driven photocatalysis. Angew Chem Int Ed.

